# Dietary glycemic index and glycemic load mediate the effect of CARTPT rs2239670 gene polymorphism on metabolic syndrome and metabolic risk factors among adults with obesity

**DOI:** 10.1186/s12902-022-01188-z

**Published:** 2022-11-21

**Authors:** Mahdieh Khodarahmi, Goli Siri, Farnoosh Erahimzadeh, Mahdieh Abbasalizad Farhangi, Dariush Shanehbandi

**Affiliations:** 1 Nutrition and Food Security Research Center, Isfahan University of Medical Sciences, Isfahan, Iran; 2 Department of Internal Medicine, Amir Alam Hospital, Tehran University of Medical Sciences, Tehran, Iran; 3 Department of Internal Medicine, Faculty of Medicine, Mashhad University of Medical Sciences, Mashhad, Iran; 4 Tabriz Health Services Management Research Center, Tabriz University of Medical Sciences, Tabriz, Iran; 5 Molecular Medicine Research Center, Tabriz University of Medical Sciences, Tabriz, Iran

**Keywords:** Glycemic index, Glycemic load, Obesity, Structural equation modeling, Metabolic syndrome

## Abstract

**Introduction:**

The importance of genetic and dietary factors in occurrence and progression of chronic diseases such as metabolic syndrome (MetS) has been established. However, complex interrelationships, including direct and indirect effects of these variables are yet to be clarified. So, our aim was to investigate the mediating role of glycemic indices in the relationship between CARTPT rs2239670 polymorphism, socio-demographic and psychological factors and metabolic risk factors and the presence of MetS in adults with obesity.

**Methods:**

In a cross-sectional study of 288 apparently healthy adults with obesity aged 20–50 years, dietary glycemic index (GI) and glycemic load (GL) were measured using a validated semi-quantitative food frequency questionnaire (FFQ). Biochemical parameters, blood pressure and anthropometric indicators were assayed by standard methods. Genotyping was carried out by polymerase chain reaction-restriction fragment length polymorphism (PCR–RFLP) technique. Structural equation modeling (SEM) was used in the statistical analysis.

**Results:**

CARTPT rs2239670 had a positive direct effect on MetS (B = 0.037 ± 0.022; *P* = 0.043) and, on the other hand, this variant was found to be indirectly associated with MetS presence through mediation of GI (B = 0.039 ± 0.017; *P* = 0.009). CARTPT was a significant predictor of both dietary GI and GL (B = 1.647 ± 0.080 and B = 3.339 ± 0.242, respectively). Additionally, glycemic indicators appeared to mediate the association of age and gender with LDL-C (B = 0.917 ± 0.332; *P* = 0.006) and HDL (B = 1.047 ± 0.484; *P* = 0.031), respectively. GI showed a positive relationship with LDL-C (*P* = 0.024) in men and similar relationships were found between GL and LDL-C (*P* = 0.050) and cholesterol (*P* = 0.022) levels in women.

**Conclusion:**

The SEM findings suggest a hypothesis of the mediating effect of glycemic indices in the relationship between genetic susceptibility to obesity and MetS presence. Our findings need to be confirmed with large prospective studies.

## Background

According to WHO, obesity is now a worldwide epidemic, with more than 1.9 billion adults overweight and 650 million of them obese. According to the latest report of WHO, more than 50% of Iranian adult are overweight or obese [1]. It is extensively related to comorbidities such as type II diabetes, cardiovascular diseases, hypertension, cancer, sleep disorders, arthritis and other musculoskeletal problems [2]. Obesity co-exists with several metabolic abnormalities, such as insulin resistance, hypertension, and dyslipidemia and it is also hypothesized to be the most common underlying cause of metabolic syndrome [3]. In fact, obesity is a complex and multifactorial disease caused by both genetic and environmental factors [4]. On the basis of scientific evidence, obesity has a genetic basis but needs environmental influence to manifest.

In recent years, identification of genetic variants contributing to obesity has become a very hot topic. The cocaine- and amphetamine-regulated transcript prepropeptide gene (CARTPT) maps to human chromosome 5q13–14, is a positional candidate for obesity [5]. This gene encodes for cocaine- and amphetamine-regulated transcript (CART) protein which is a neuropeptide involved in many physiological processes, especially those controlling feeding behavior and body weight [6]. This peptide is co-expressed with proopiomelanocortin (POMC) neurons in the arcuate nucleus of the hypothalamus. Indeed, alpha melanocyte-stimulating hormone (α-MSH) which is produced from the POMC precursor, along with CART inhibit food intake and increase energy expenditure [7]. Since genetic variations in CARTPT may influence expression and/or function of the peptide, they can influence susceptibility to diseases/disorders. There is accumulating evidence showing that polymorphisms in the CARTPT gene are associated with obesity [8]. However, the findings in this regard remain inconclusive and controversial [9]. It’s hard to explain these conflicting results from the biological viewpoint. However, the specific environmental factors particularly diet that interact with obesity-predisposing gene variants may contribute to these inconsistencies and modulate the effects of CARTPT gene polymorphisms on obesity and its-related diseases.

Diet, as a major modifiable determinant of obesity, plays an important role in the development and the prevention of obesity-related comorbidities. Among dietary factors contributing to obesity risk, carbohydrate intake is of great importance, particularly in countries such as Iran, where high carbohydrate foods are the main source of energy intake. In this regard, it has been reported that Iranian adults receive more than 60% of their energy intake from carbohydrates, in particular refined grains which are mainly associated with high dietary glycemic index (GI) and glycemic load [10]. Although a number of studies have reported the beneficial effects of low dietary GI and GL on obesity [11], cardio-metabolic risk factors and MetS, the results of other studies in this regard are inconsistent [12, 13]. For example, several different meta-analyses of randomized controlled trials (RCTs) have indicated that low-GI or GL diets lead to a significant greater reduction in fasting blood glucose, glycated hemoglobin [14], total cholesterol and low density lipoprotein cholesterol (LDL-c) [15] compared with control diets. On the other hand, some earlier observational studies have found that diets with a high GI and GL are associated with greater risks of MetS or its components [16]. However, this potential protection that Low-GI/GL diets offer against MetS has not been confirmed by a recent meta-analysis [17] and some prospective studies [18]. On the other hand, the associations of different socio-demographic (age, socio-economic and marital statuses) [19] and psychological variables [20] with the development of obesity have been established in previous studies and these associations may be mediated through dietary intakes. For instance, lower socioeconomic status is associated with an unhealthy diet which in turn leads to a higher risk of adiposity and its-related health outcomes [21]. Moreover, it has been shown that obesity and its-related complications are influenced not only by all of abovementioned factors, but also by their interactions in interconnected biological pathways or networks [22]. However, the most studies have focused only on the relationship between a limited number of independent variables and a single outcome and a very few studies have considered simultaneously a large various set of determinants of obesity-associated metabolic complications [23]. On the other hand, whether unhealthy eating is a mediating factor in the association of all of these determinants with obesity and its-related health outcomes is still unknown.

Structural equation modeling (SEM) is a comprehensive and powerful multivariate analysis technique which allows us to conceptualize the structure of predisposing factors of obesity-associated metabolic complications as a model and simultaneously analyze all complex interrelationships between inter-dependent variables as relevant regression pathways [24] To our knowledge, no evidence is available on the simultaneous direct and indirect associations between modifiable risk factors and MetS as a system of multiple pathways. Therefore, the aim of the present study was to determine the direct and indirect associations of potential genetic and socio-demographic factors and dietary glycemic indices with metabolic risk factors and MetS among adults with obesity.

## Methods

### Participants

Between November 2017 and October 2018, a total of 288 apparently healthy adults with obesity were recruited in this cross-sectional study using convenience sampling method through announcements and flyers placed in public places and health care facilities in Tabriz city, one of the biggest cities of Iran. These announcements provided general information about inclusion criteria (age 20 to 50 years, good health and obesity (BMI ≥ 30 kg/m^2^)). At first, 350 individuals applied to participate in research. However, after eligibility screening according to the inclusion and exclusion criteria, 60 subjects were removed from the study. Exclusion criteria were as follows: being pregnant and lactating, experiencing pregnancy, lactation, and menopause, current smokers, having any evidence of chronic disease such as cardio-vascular diseases, hypertension, hyperlipidemia, diabetes, renal diseases, hepatic disorders, and cancer, use of any medications and supplements affecting weight and variables studied such as loop diuretics, cortico-steroids, antidepressants, statins and antihypertensive agents. As well as, individuals who had any recent surgery such as bariatric were excluded. Furthermore, individuals (*n* = 2) who reported total calorie intake outside the range of 800–4200 kcal/d were also excluded due to under- and over-reporting of energy intake [25]. With regard to the maximum RMSEA of 0.08 [26], α = 0.05 and power of 80%, by using statistica software (version 10), the minimum sample size was calculated to be 184. Overall, 288 participants who agreed to take part were analyzed. All of the participants completed a written informed consent prior to participation in the study. The procedures of this study were approved by the Ethical Committee of the Tabriz University of Medical Sciences (registration code: IR.TBZMED.REC.1399.207). The presence of MetS was identified according to the National Cholesterol Education Program (NCEP) Adult Treatment Panel (ATP) III criteria [27]. Based on this definition, the presence of three or more of the following criteria was considered to be MetS: abdominal obesity (waist circumference (WC) > 102 cm (men) or > 88 cm (women)), systolic blood pressure ≥ 130 or diastolic blood pressure ≥ 85 mmHg, fasting blood sugar ⩾110 mg/dl, fasting triglyceride (TG) level ⩾150 mg/dl, fasting high-density lipoprotein (HDL) cholesterol level < 40 mg/dl for men or 50 mg/dl for women.

### Dietary intake assessment and glycemic indices calculation

The usual dietary intakes of the participants were assessed by using a reliable [28] and validated [29] 147-item semi-quantitative food frequency questionnaire (FFQ) through face to face interview by an expert interviewer. Age and energy-adjusted correlation coefficients between mean carbohydrate intakes of the 24 h dietary recalls and FFQ were 0.39 and 0.47 in men and women, respectively [29]. Participants were asked to report their frequency and amount of the intake of given food items during the last year on a daily, weekly, or monthly basis and then these reported portion sizes were converted to grams using household measurements. Daily nutrient intakes were estimated based on Iranian Food Composition Table (FCT) [30] and missing information were complemented according to the United States Department of Agriculture FCT [31]. GI, an indicator of dietary carbohydrate quality, quantifies the postprandial blood glucose and insulin responses to carbohydrate composition of the certain meals [32], and the concept of GL represents both the GI and the quantity of carbohydrate intake [33]. With glucose as a reference scale, total dietary GI was calculated by using the following formula: ∑(GI_a_ ˟ available carbohydrate_a_) /total available carbohydrate where available carbohydrate of food items was calculated as total carbohydrate minus dietary fiber [34]. In this equation, GI_a_ = GI of the a^th^ food and available carbohydrate_a_ was grams of available carbohydrate in the a^th^ food [34]. Of the 147 food and drink items included in the questionnaire, 100 items were available carbohydrate containing foods. GI values of each carbohydrate-containing food item were derived from Iranian food table [35]. Since Iranian national table does not cover the GI of all available foods, GI of unrecorded foods was derived from international references [36, 37]. Dietary glycemic load was calculated as (total GI ˟ total available carbohydrate)/100 [34].

### Socio-demographic, anthropometric and blood pressure assessments

Socio-demographic data including age, gender, marital status, smoking and the history of any diseases were asked by an expert interviewer. Socioeconomic status was assessed through the following questions: occupation, educational status, family size and home ownership as individual indicators. Education was divided into six categories depend on the highest level of educational attainment: illiterate: 0, less than diploma: 1, diploma and associate degree: 2, bachelors: 3, masters: 4 and higher: 5. Occupational status of male participants was hierarchically categorized as unemployed: 0, worker, farmer and rancher: 1, others: 2, employee: 3 and self-employed: 4. Female subjects of different professions were grouped into housewife, employee, student, self-employed and others. Each subject was asked to answer that his/her house ownership belongs to which of two categories defined as: tenant or landlord. Additionally, family size variable was categorized as: ≤3, 4–5, ≥6. Subsequently, scores of each item summed and the whole SES score was computed with a range of zero to 16. After calculating overall score, individuals were categorized into 3 categories: low, middle, and high based on SES tertiles. International Physical Activity Questionnaire was implemented to assess the physical activity level of participants [38]. Height and weight were measured while the participant stood in light clothing and in bare foot using a tape measure and Seca scale (Seca, Germany) to the nearest of 0.1 cm and 100 g, respectively. Body mass index (BMI) of participants was calculated by dividing the body weight by height in meters squared (kg/m^2^). Waist circumference (WC) was measured at the narrowest area of the waist and at the end of normal exhalation by a stretch-resistant tape measure with accuracy of 0.1 cm [39]. Systolic blood pressure (SBP) and diastolic blood pressure (DBP) were measured using a standardized mercury sphygmomanometer after at least 15 min rest in a sitting position, and the average of the two measurements was recorded [23].

### Mental health and appetite assessment

In order to determine the severity of the emotional disturbance of participants, a self-administered the Depression, Anxiety and Stress Scale-21 Items (DASS-21) questionnaire was applied which has been validated for using in the Iranian population [40, 41]. The Cronbach’s α coefficients (reliability) for the DASS questionnaire in Iranian subjects have been reported as 0.77, 0.79 and 0.78 for depression, anxiety and stress, respectively [40]. This questionnaire consists of three categories of 7-item self-report scales (Depression, Anxiety, and Stress) which uses the Likert four-level scoring system ranging from zero (“did not apply to me at all”) to 3 (“applied to me very much or most of the time”). The total score for each category was determined by summing the scores for the relevant items and then multiplied by 2 with a range of 0 to 21 for each subscale. According to the DASS cut-off scores by Lovibond and Lovibond, individuals were categorized into 5 categories: normal, mild, moderate, severe and extremely severe [42]. Higher scores indicated a greater severity of psychological symptoms.

To assess appetite sensations, a 10 cm visual analog scale (VAS) questionnaire (about hunger, fullness, satiation, desire to eat sweet/salty/fatty foods, and prospective food consumption) was used. This questionnaire which was validated in previous studies [43] was completed by making a mark on each 100 mm line corresponding to the feeling of participants. Each score was determined by measuring millimeters from the left side of the line to the mark.

### Biochemical analysis

After 12-hour overnight fasting, venous blood samples were collected from all the subjects. Blood samples were centrifuged at 4500 rpm for 10 min at 4 °C and serums were immediately separated and stored at − 80 C until analysis. Serum total cholesterol (TC), glucose, triglyceride (TG), serum high-density lipoprotein (HDL) cholesterol were determined by commercial kit (Pars Azmoon, Tehran, Iran). Serum level of insulin was measured using commercially available enzyme-linked immunosorbent assay (ELISA) kits (Bioassay Technology Laboratory, Shanghai Korean Biotech, Shanghai City, China). Low-density lipoprotein cholesterol (LDL-C) was estimated according to the Friedewald method [44]. We also calculated homeostasis model assessment-insulin resistance index (HOMA-IR) and quantitative insulin sensitivity check index (QUICKI) based on the protocols by Matthews et al. [45] and Katz et al. [46], respectively. The atherogenic index of plasma (AIP) was defined as the base 10 logarithm of TG to HDL ratio [47].

### Genetic analysis

The genomic DNA of participants was extracted from the whole blood using a standard phenol/chloroform method. Subjects were genotyped for the CARTPT rs2239670 (major allele: G; minor allele: A) polymorphism using polymerase chain reaction-restricted length polymorphism (PCR–RFLP) technique. Template primers used for the PCR amplification of the rs2239670 were as follow: forward: CCTGCTGCTGATGCTACCTCT-3′ and reverse: 5′-GCGCTTCGATCTGCAACACAC-3′. The PCR reaction was optimized in a total volume of 25 μl containing 0.5 μl of each primer, 2 μl of DNA, 10 μl of Taq DNA Polymerase 2 × MasterMix (Ampliqon, Denmark) and 12.5 μl distilled water. For PCR amplification, the following conditions were applied: 94 °C/5 min (initial denaturation), 35 cycles of denaturation 94 °C/30 s, annealing 60 °C/30 s, extension 72 °C/20 s and 72 °C/10 min (final synthesis). Digestion of the amplified DNA was performed with ApaI (Takara, Japan) restriction enzyme overnight. The digested products were then analyzed by electrophoresis on 3% agarose gel. Homozygous for wild-type allele (GG) of the CARTPT rs2239670 was distinguished as cut fragments (340 and 212 bp), heterozygous as cut fragments (212, 340 and 552 bp) and homozygous for mutant allele (AA) as uncut fragment (552 bp).

### Statistical analysis

**Fig. 1:**
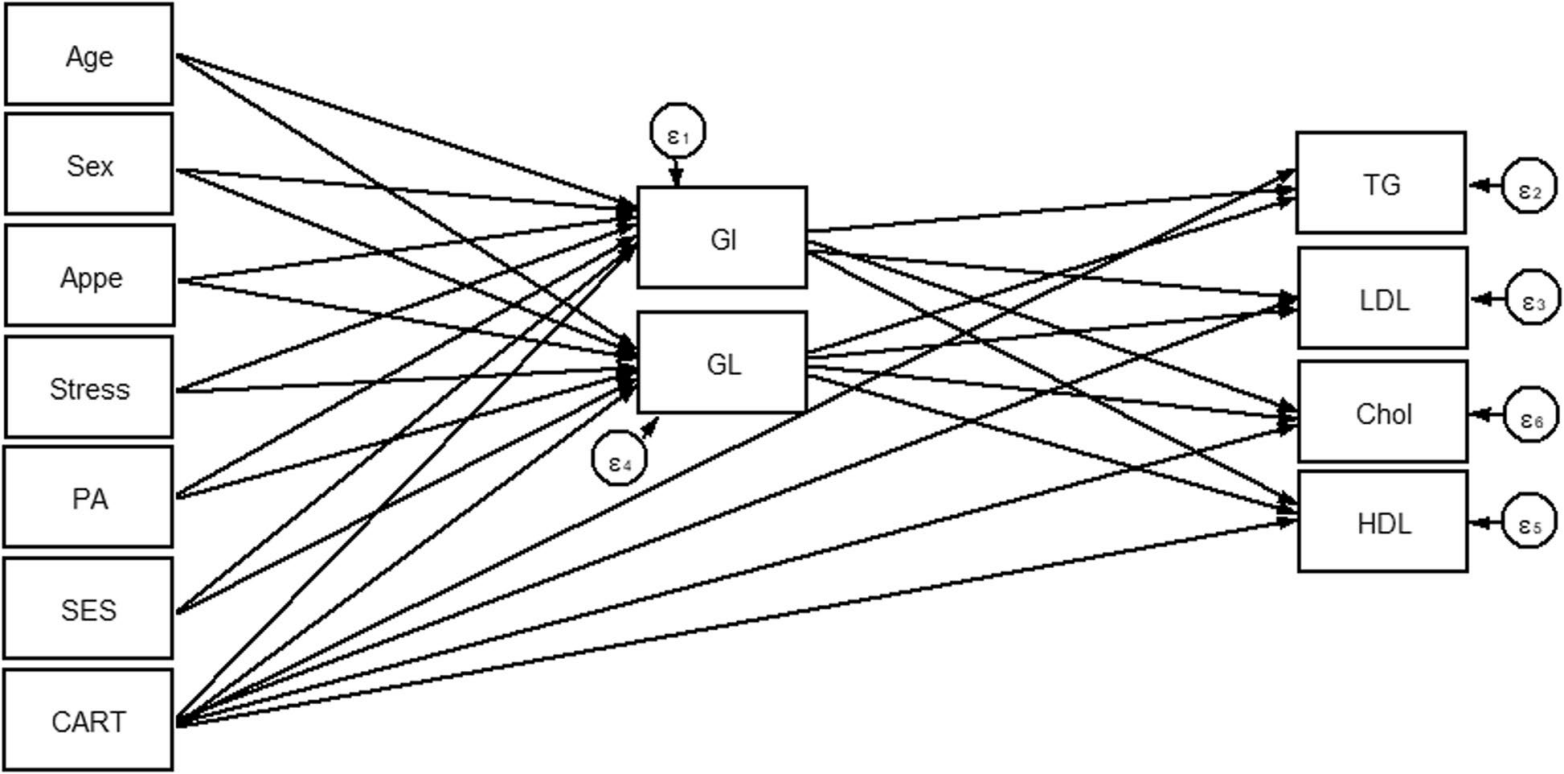
Hypothesized models in which GI and GL as mediating variables relate CARTPT rs2239670 polymorphism, socio-demographic and psychological parameters to serum lipids. Abbreviations: CART, cocaine- and amphetamine-regulated transcript; GI, glycemic index; GL, glycemic load; SES, socio-economic status; PA, Physical activity; Appe, appetite; LDL-C, low density lipoprotein cholesterol; HDL, high-density lipoprotein; TG, triglyceride; Chol, cholesterol.

**Fig. 2:**
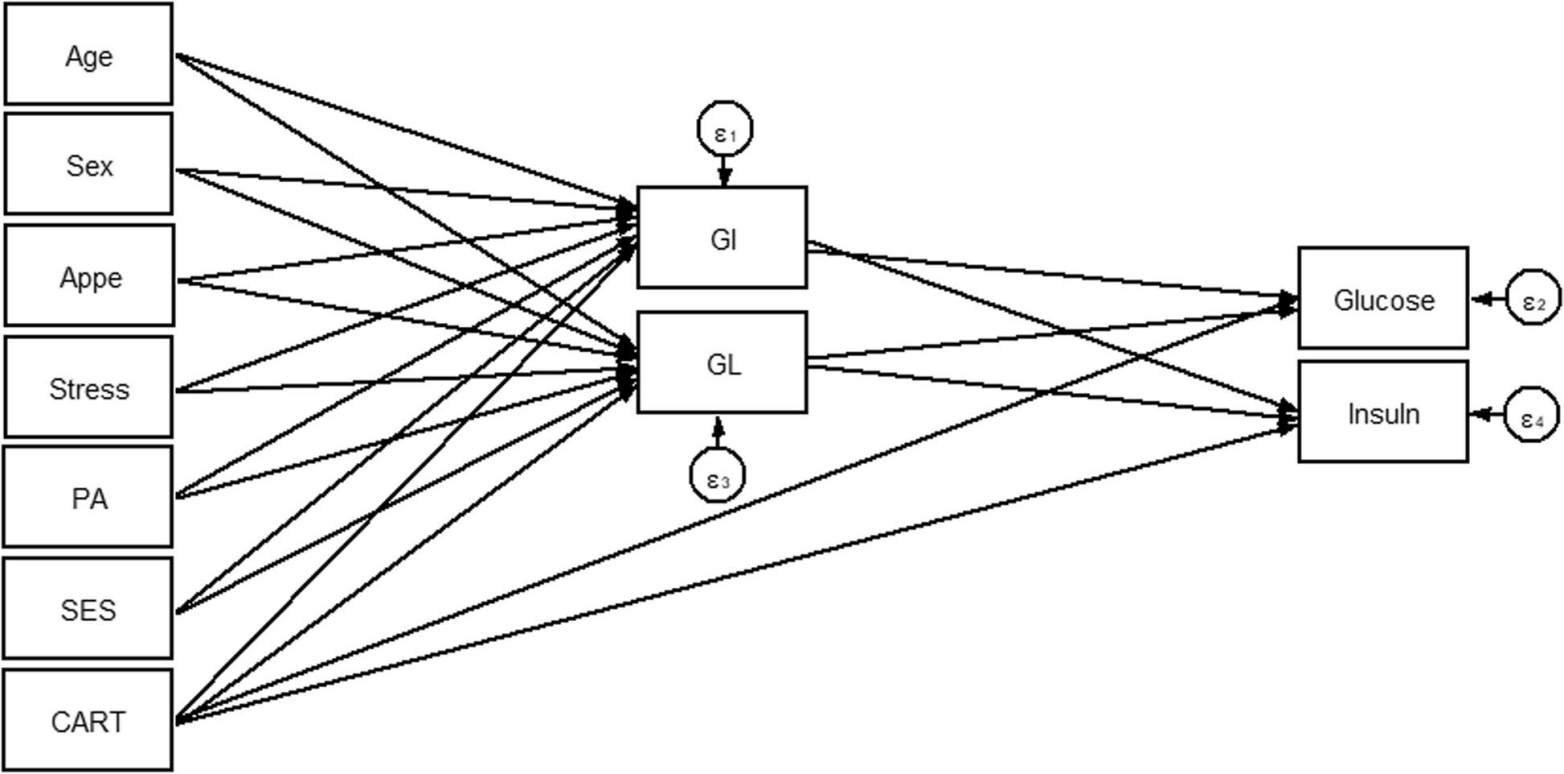
Hypothesized models in which GI and GL as mediating variables relate CARTPT rs2239670 polymorphism, socio-demographic and psychological parameters to serum glycemic levels. Abbreviations: CART, cocaine- and amphetamine-regulated transcript; GI, glycemic index; GL, glycemic load; SES, socio-economic status; PA, Physical activity; Appe, appetite.

**Fig. 3:**
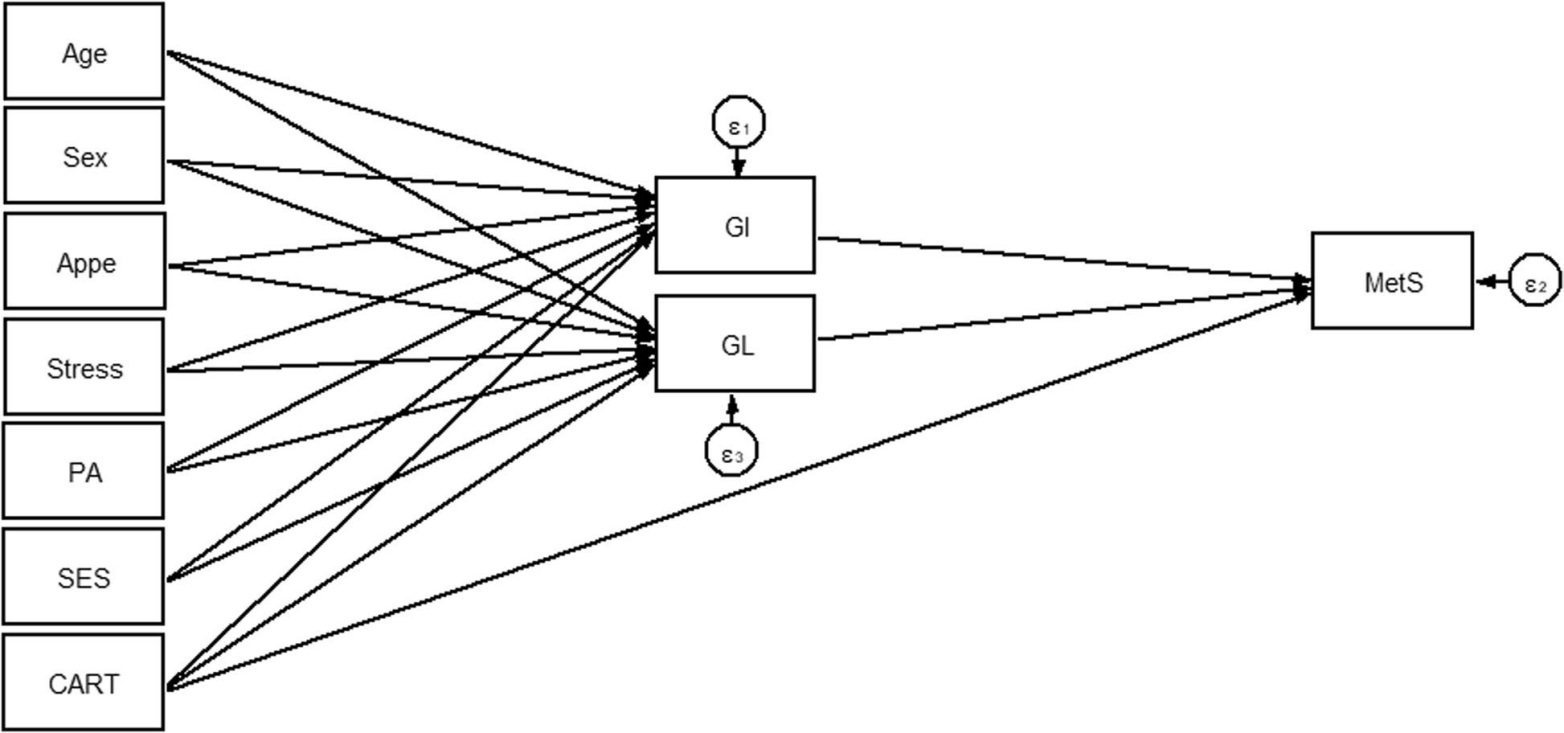
Hypothesized models in which GI and GL as mediating variables relate CARTPT rs2239670 polymorphism, socio-demographic and psychological parameters to MetS. Abbreviations: CART, cocaine- and amphetamine-regulated transcript; GI, glycemic index; GL, glycemic load; SES, socio-economic status; PA, Physical activity; Appe, appetite; MetS, metabolic syndrome.

The normality of data was tested by descriptive measures such as coefficients of skewness and kurtosis, mean and standard deviation [48] and all parameters except TG, glucose, HOMA-IR and insulin were normally distributed. For the descriptive analysis, the data were presented as mean ± standard deviation (SD) for normally distributed continuous variables, the median (25 and 75 percentiles) for variables with skewed distributions, and the frequency (%) for categorical variables. The GI and GI scores were classified into tertiles according to gender groups. The comparison of categorical and continuous variables between different tertiles of GI and GL scores was performed by Chi- square test and analysis of variance (ANOVA), respectively. SEM analysis was carried out to examine the proposed conceptual models, namely, the mediating effect of dietary glycemic indices on the role of genetic susceptibility, socio-demographic variables and mental health in metabolic risk factors and also the presence of MetS. These conceptual models (shown in Figs. 1, 2 and 3) were determined based on theory, logical grounds and previous studies. In the conceptual models 1 and 2, dietary GI and GL were expected to be directly related to each socio-demographic [49], genetic [50] and psychological factors [51] and, on the other hand, the indirect effects of these mentioned parameters on lipid profile [52] and serum glycemic levels [53] were mediated by GI and GL. Accordingly, in the conceptual model 3, MetS was considered as an endogenous variable which could be influenced by exogenous variables, namely, dietary [17], socio-demographic [54], genetic [55] and psychological factors [56]. SEM is a powerful multivariate analysis technique that often includes two important stages, the measurement model (estimation of the effects of unobserved or latent constructs), which was not applicable for the present study, and the structural model (path analysis which examines the relationships between latent constructs and other observed variables) [24]. In the current study, several path analyses, which estimate the direct and indirect associations between exogenous and endogenous variables, were run for 2 specific purposes: 1) to identify if the association between socio-demographic, genetic determinants, mental health and cardio-metabolic risk factors are mediated by glycemic indices and 2) to test whether dietary glycemic indices mediate the genetic susceptibility to MetS. Generally, after developing conceptual model, identification of model was assessed. In the next step, the method of maximum likelihood was applied to estimate regression coefficients when the outcome variables met a normal distribution. Modification indices were examined and implemented in order to determine whether conceptually appropriate changes could be made to improve model fit or not. Fitting of conceptual models to data was evaluated using following goodness-of-fit indices: chi-square test (χ^2^/ degrees of freedom (df) ratio < 5 [57], comparative fit index (CFI) > 0.90 [58], standardized root mean square residual (SRMR) < 0.08 [59], and root mean square error of approximation (RMSEA) ≤0.08 [59]. Data management and all statistical analyses were conducted using STATA version 14.2 and Mplus software (version 7.4; Muthén & Muthén). In all analyses, *P*-values< 0.05 were considered statistically significant.

## Results

**Fig. 4:**
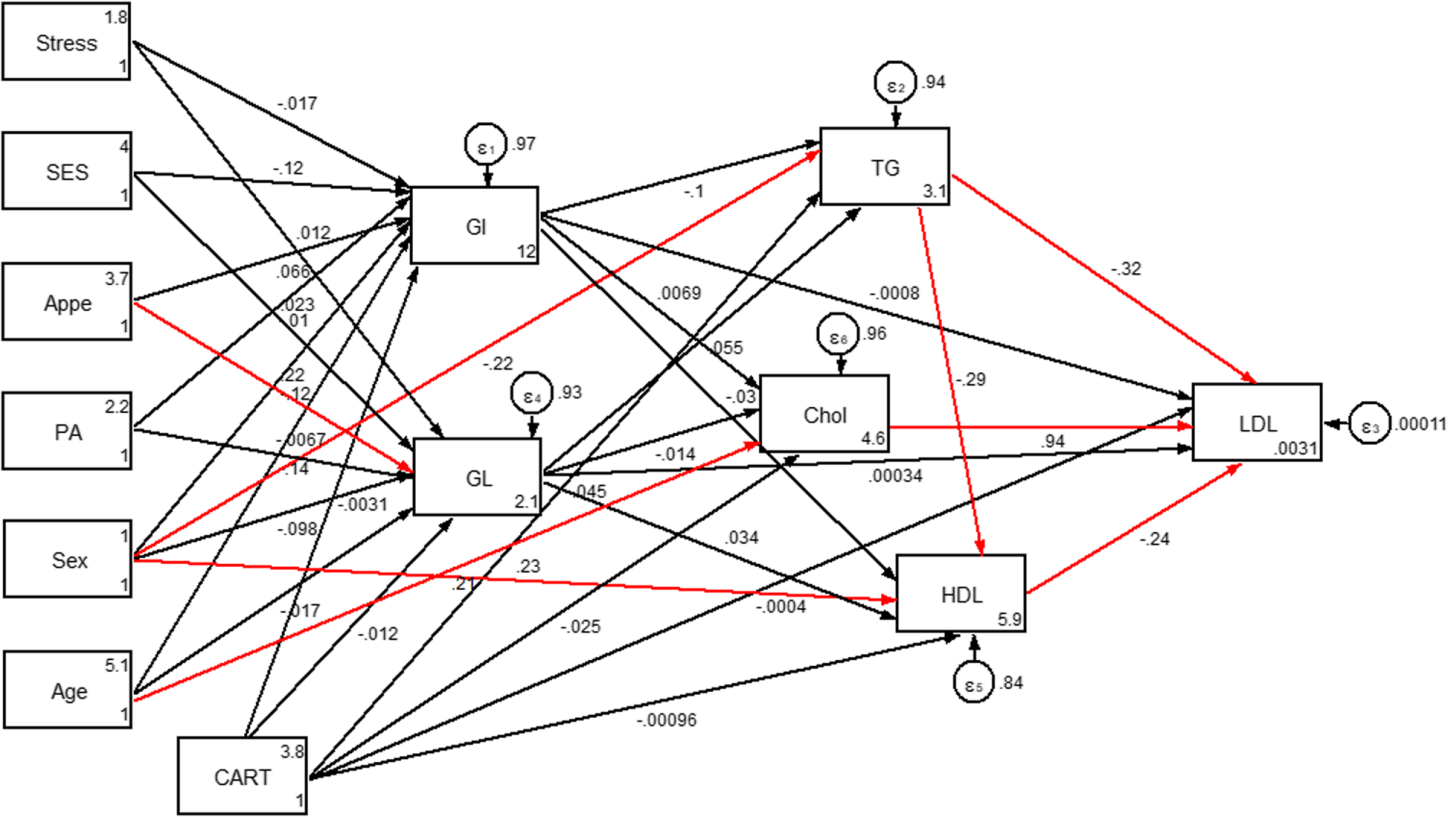
Path analysis diagram with standardized estimates illustrating the total effects of CARTPT rs2239670 polymorphism, diet, socio-demographic and psychological parameters on lipid profile among adults with obesity. Abbreviations: CART, cocaine- and amphetamine-regulated transcript; GI, glycemic index; GL, glycemic load; SES, socio-economic status; PA, Physical activity; Appe, appetite; LDL-C, low density lipoprotein cholesterol; HDL, high-density lipoprotein; TG, triglyceride; Chol, cholesterol.*All path coefficients are standardized. Red arrows mean *p*.value ≤0.05. £Total effect is defined as the sum of direct and indirect effects.

**Fig. 5:**
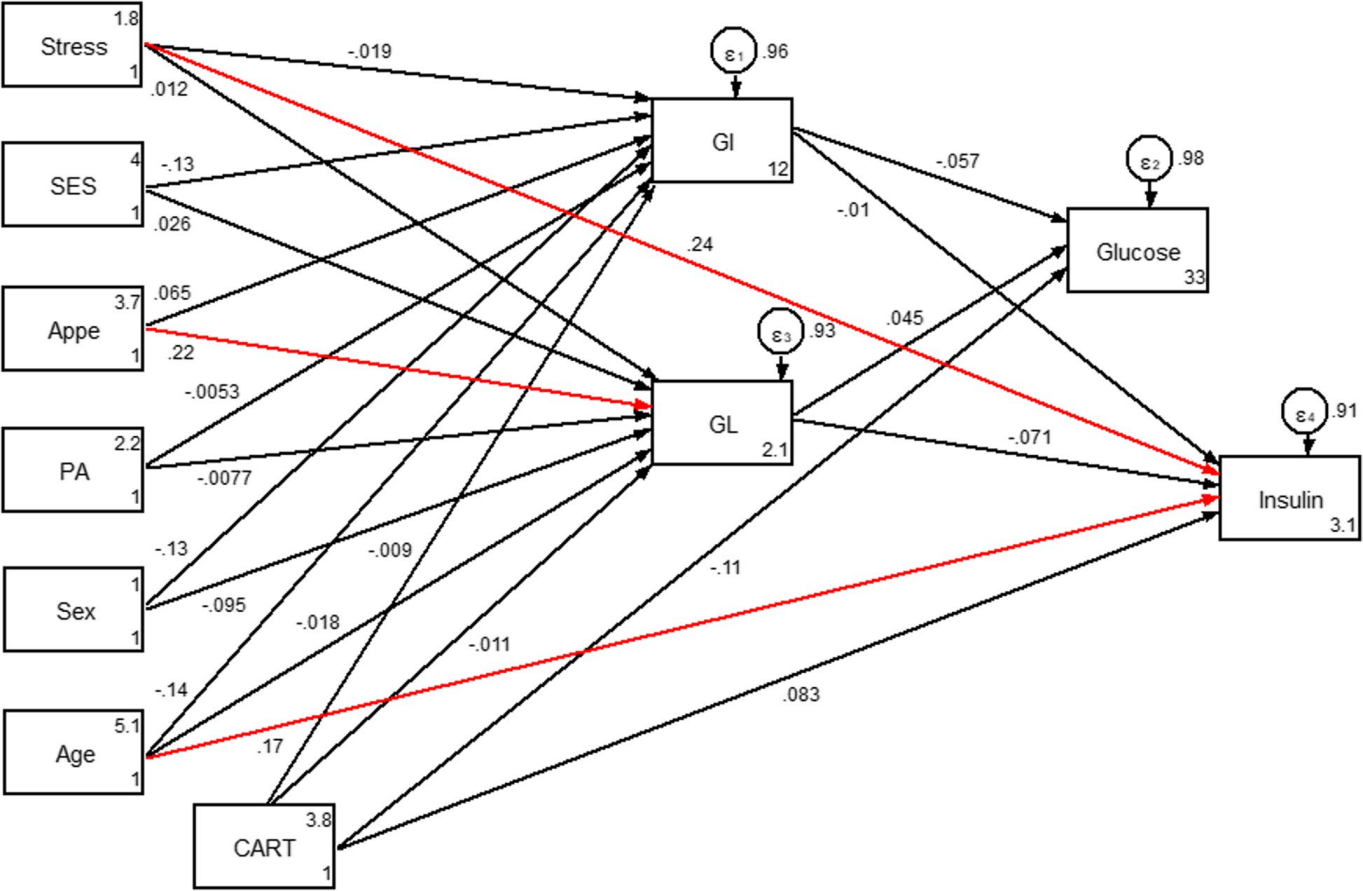
Path analysis diagram with standardized estimates illustrating the total effects of CARTPT rs2239670 polymorphism, diet, socio-demographic and psychological parameters on serum glycemic levels among adults with obesity. Abbreviations: CART, cocaine- and amphetamine-regulated transcript; GI, glycemic index; GL, glycemic load; SES, socio-economic status; PA, Physical activity; Appe, appetite *All path coefficients are standardized. Red arrows mean *p*.value ≤0.05. £Total effect is defined as the sum of direct and indirect effects

**Fig. 6:**
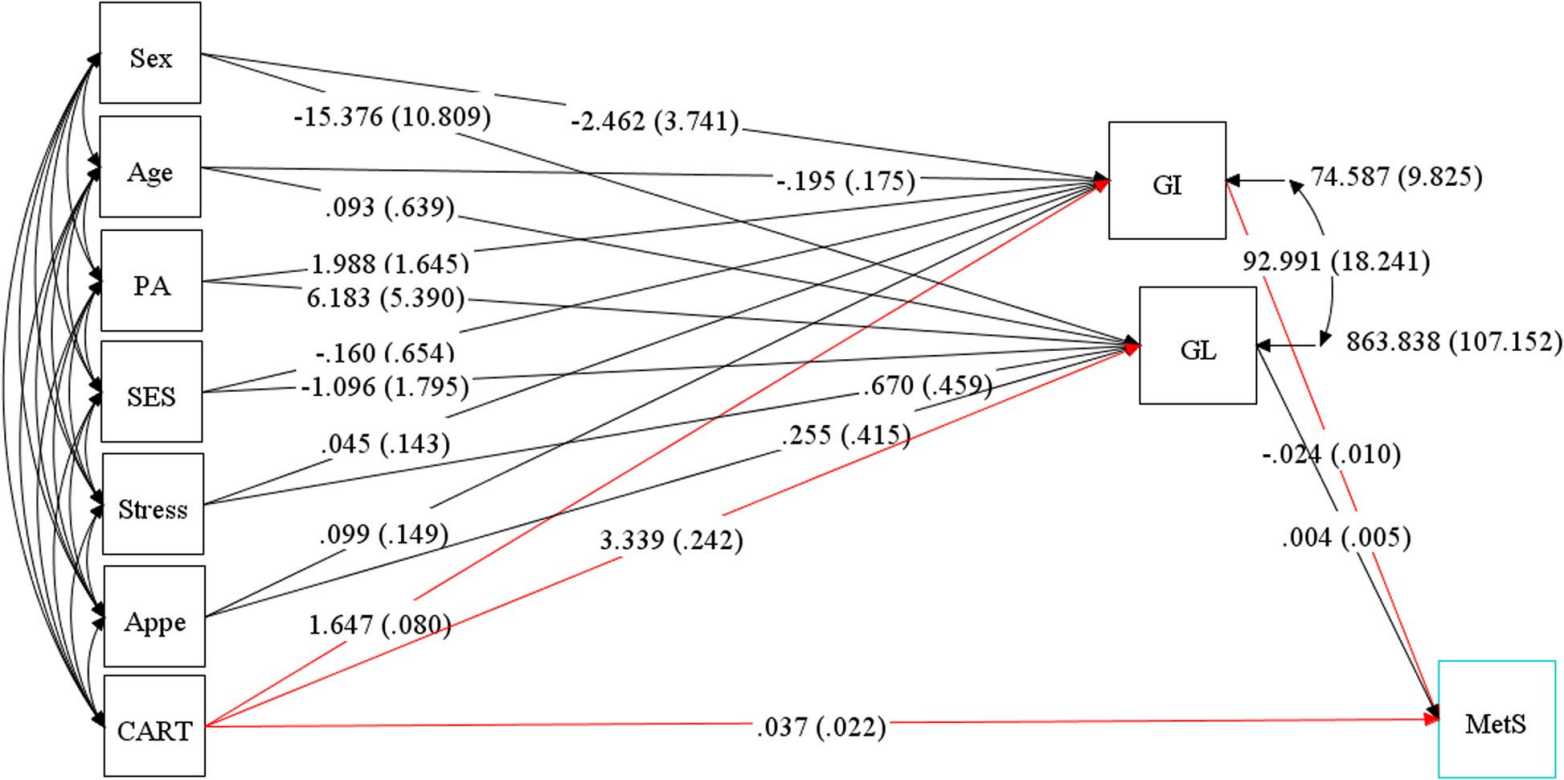
Structural equation model diagram with standardised estimates for total effects of genetic, socio-demographic and psychological parameters and diet on metabolic syndrome among adults with obesity. Abbreviations: CART, cocaine- and amphetamine-regulated transcript; GI, glycemic index; GL, glycemic load; SES, socio-economic status; MetS, metabolic syndrome; PA, Physical activity; Appe, appetite. *All path coefficients are standardized. Red arrows mean *p*.value ≤0.05. ^£^Total effect is defined as the sum of direct and indirect effects

Overall, 288 participants with complete information were included in the analyses. Socio-demographic, genetic, psychological and metabolic parameters of participants across GI and GL tertiles among men and women are presented in Tables 1 and 2, respectively. No significant differences in terms of anthropometric, socio-demographic and mental health parameters across tertile categories of GI and GL were seen; neither in women nor in men. Among male subjects, a high dietary GI was positively associated with higher LDL-C levels (*P* = 0.024). Similarly, among female subjects, there were significant associations between dietary GL and LDL-C (*P* = 0.050) and cholesterol (*P* = 0.022) concentrations. The direct and indirect effects of the genetic, socio-demographic and psychological variables through dietary glycemic indices (GI and GL) on lipid profile (model 1) and serum glycemic levels (model 2) were evaluated and significant standardized path coefficients are presented in Table 3. As shown in Table 3, in the model 1, gender played an important role, with a strong indirect positive effect on HDL (B = 1.047; *P* = 0.031) through mediatory effects of GI and GL. However, the direct effect of gender on HDL (B = 3.885; *P* = 0.002) was larger than its indirect effects. The direct effect of age on cholesterol (B = 0.938; *P* = 0.005) was as strong as its indirect effects on LDL-C (B = 0.917; *P* = 0.006). Besides, gender was the largest contributing variable to TG (B = − 24.740; *P* = 0.004). The direct effect of appetite on GL was strong as well (B = 1.358; *P* = 0.003). There was no significant direct or indirect relationship between genetic variant (CARTPT rs2239670) and serum glycemic levels and also lipid profile in both models (models 1 and 2). In the model 2, stress (B = 0.007; *P* = 0.001) and age (B = 0.006; *P* = 0.023) had positive direct effects, but not as strong, on the insulin levels, whereas no significant indirect relationship was found in this model. The goodness-of-fit indices for models 1 and 2 were: χ^2^/d.f. = 1.99; RMSEA = 0.075 (90% CI = 0.043, 0.106); CFI = 0.985 and χ^2^/d.f. = 1.90; RMSEA = 0.072 (90% CI = 0.023, 0.116); CFI = 0.901, respectively; indicating a satisfactory model fit to data (Table 4). Figures 4 and 5 show the path analysis diagrams with standardized estimates for the relationships of socio-demographic, genetic and psychological parameters and diet with serum lipid profile and glycemic levels, respectively. As hypothesized, there was a significant positive direct, but weak, effect of CARTPT on MetS (B = 0.037; *P* = 0.043) (model 3, Table 3). On the other hand, a weak positive indirect association was found between this variant and MetS which specifically was mediated through GI (B = 0.039; *P* = 0.009). Total effects of study variables on MetS are displayed as path diagram in Fig. 6 and Table 5. CARTPT (B = 0.037, *P* = 0.04) and higher GI score (B = 0.024, *P* = 0.008) were positively associated with the MetS presence. Additionally, CARTPT was a strong predictor of both dietary GI (B = 1.647, *P* < 0.05) and GL (B = 3.339, *p* < 0.05). Fit indices of the model 3 also indicated acceptable fit thresholds (χ^2^/df = 1.28; CFI = 0.997; RMSEA (95% CI) = 0.055 (0.000–0.152)).

**Table 1:** Socio-demographic and anthropometric characteristics and cardio-metabolic risk factors according to the tertiles of dietary glycemic index in men

	Glycemic index				Glycemic load				
	T1	T2	T3	P*		T1	T2	T3	P*
**Age (y)**	39.25 (5.76)	39.03 (7.48)	36.91 (6.37)	0.387	39.96 (6.31)		36.86 (6.80)	38.65 (6.42)	0.234
**WC**	108.75 (9.29)	114.72 (5.81)	112.54 (6.94)	0.475	112.39 (5.29)		114.16 (8.92)	112.69 (7.05)	0.588
**BMI (kg/m**^**2)**^	33.57 (4.06)	34.08 (2.53)	33.73 (2.86)	0.708	33.19 (2.59)		34.11 (3.67)	33.87 (2.96)	0.559
**Physical activity level, (%)**				0.207					0.461
Low	41.2	29.4	29.4		20.6		47.1	32.4	
Moderate	28.1	25.0	46.9		28.1		34.4	37.5	
High	20.0	46.7	33.3		23.3		26.7	50.0	
**Marital status, (%)**				0.479					0.554
Married	20.0	20.0	40.0		13.3		46.7	40.0	
Single	32.1	32.1	35.8		25.9		34.6	39.5	
**SES, n (%)**				0.450					0.468
Low	0.0	0.0	0.0		0.0		0.0	0.0	
Middle	16.7	50.0	33.3		26.7		23.3	50.0	
High	35.3	26.2	38.5		23.1		43.1	33.8	
**Stress, n (%)**				0.846					0.171
Normal	30.4	37.0	32.6		19.6		45.7	34.8	
Mild	29.4	35.3	35.3		47.1		23.5	29.4	
Moderate	29.4	23.5	47.1		35.3		17.6	47.1	
Severe	20.0	40.0	40.0		0.0		30.0	70.0	
Extremely severe	50.0	16.7	33.3		0.0		66.7	33.3	
**Appetite**	35.14 (9.29)	34.83 (9.94)	34.80 (9.73)	0.883	31.04 (9.67)		35.89 (9.72)	36.53 (8.84)	0.110
**LDL-C, (mg/dl)**	112.60 (25.50)*	118.12 (24.27)	129.12 (29.34)*	**0.024**	119.20 (27.43)		125.14 (28.04)	116.94 (26.57)	0.221
**HDL, (mg/dl)**	42.43 (7.50)	42.90 (8.69)	42.57 (7.62)	0.986	41.52 (7.75)		43.89 (8.93)	42.09 (6.71)	0.474
**Cholesterol, (mg/dl)**	185.21 (33.07)	184.28 (26.74)	197.86 (31.71)	0.084	184.00 (31.07)		197.69 (31.65)	185.41 (29.44)	0.095
**TG, (mg/dl)**	125.50 (96.50, 177.50)	116.00 (87.50, 134.50)	111.00 (78.00, 169.00)	0.214	90.00 (80.00, 134.00)		121.00 (92.00, 159.00)	123.00 (87.50, 169.00)	0.204
**AIP**	0.14 (0.23)	0.05 (0.23)	0.08 (0.27)	0.229	0.04 (0.26)		0.11 (0.25)	0.10 (0.23)	0.511
**Glucose, (mg/dl)**	92.00 (85.00, 99.25)	91.00 (86.50, 100.00)	91.00 (85.00, 101.00)	0.985	91.00 (85.00, 100.00)		92.00 (89.00, 101.00)	91.50 (84.00, 101.00)	0.844
**Insulin, U/mL**	15.30 (9.15, 26.60)	10.60 (8.05, 18.20)	11.50 (9.00, 17.20)	0.170	12.20 (10.00, 23.10)		13.20 (8.60, 23.60)	10.80 (8.75, 18.32)	0.473
**HOMA-IR**	3.58 (2.03, 5.97)	2.68 (1.75, 4.25)	2.70 (1.94, 4.00)	0.267	3.22 (2.10, 5.25)		3.20 (1.95, 4.88)	2.46 (1.86, 3.94)	0.452
**QUICKI**	0.32 (0.03)	0.33 (0.03)	0.33 (0.03)	0.371	0.32 (0.03)		0.32 (0.03)	0.33 (0.03)	0.493
**SBP (mmHg)**	112.21 (22.87)	117.93 (13.79)	118.71 (14.11)	0.286	117.17 (12.78)		120.29 (12.66)	112.12 (22.53)	0.217
**DBP (mmHg)**	71.75 (16.08)	78.10 (9.77)	77.14 (11.90)	0.108	77.61 (13.56)		76.71 (10.64)	73.65 (14.56)	0.486
**Mets (%)**	32.4	29.7	37.8	0.936	24.3		35.1	40.5	0.953
**CART (%)**				0.432					0.432
AA	3.6	10.0	3.4		0.0		6.1	9.1	
AG	25.0	23.3	13.3		13.6		21.2	24.2	
GG	71.4	66.7	83.3		86.4		72.7	66.7	

Data are presented as mean (SD) or median (25 and 75 percentiles)

*Analysis of variance for continuous variables and χ^2^ test for categorical variables

*Abbreviations*: *BMI* Body mass index, *WC* Waist circumference, *SES* Socio-economic status, *HOMA-IR* Homeostasis model assessment of insulin resistance, *LDL-C* Low density lipoprotein cholesterol, *HDL* High-density lipoprotein, *SBP* Systolic blood pressure, *DBP* Diastolic blood pressure, *TG* Triglyceride, *QUICKI* Quantitative insulin sensitivity check index, *AIP* Athrogenic indx of plasma

**Table 2:** Socio-demographic and anthropometric characteristics and cardio-metabolic risk factors according to the tertiles of dietary glycemic index in women

	Glycemic index				Glycemic load				
	T1	T2	T3	P*		T1	T2	T3	P*
**Age (y)**	38.85 (8.52)	38.13 (7.93)	36.81 (8.29)	0.570	38.90 (8.43)		36.57 (5.76)	38.08 (9.96)	0.477
**WC**	104.11 (11.39)	103.64 (10.46)	105.81 (8.09)	0.805	102.07 (9.60)		105.30 (11.40)	107.40 (8.95)	0.113
**BMI (kg/m**^**2)**^	35.36 (4.59)	35.94 (4.28)	35.72 (3.91)	0.865	35.07 (4.36)		36.19 (4.47)	36.04 (3.87)	0.570
**Physical activity level, n (%)**				0.668					0.467
Low	32.1	39.3	28.6		37.5		30.4	32.1	
Moderate	50.0	10.0	40.0		65.0		25.0	10.0	
High	37.5	37.5	25.0		37.4		31.3	31.3	
**Marital status, n (%)**				0.489					0.301
Married	63.6	18.2	18.2		36.3		18.2	45.5	
Single	31.7	35.4	32.9		44.3		30.4	25.3	
**SES, n (%)**				0.064					0.298
Low	0.0	20.0	80.0		40.0		20.0	40.0	
Middle	36.2	36.2	27.5		47.8		29.0	23.2	
High	50.0	22.2	27.8		27.8		33.3	38.9	
**Stress, n (%)**				0.375					0.877
Normal	40.0	40.0	20.0		43.3		26.7	30.0	
Mild	40.0	26.7	33.3		46.7		20.0	33.3	
Moderate	33.3	33.3	33.3		48.1		40.7	11.2	
Severe	29.4	23.5	47.1		29.4		29.4	41.2	
Extremely severe	66.7	33.3	0.0		66.7		0.0	33.3	
**Appetite**	31.68 (10.19)	32.63 (7.80)	33.11 (5.67)	0.766	31.40 (8.83)		34.46 (6.45)	31.92 (8.73)	0.299
**LDL-C, (mg/dl)**	127.12 (40.14)	116.93 (25.42)	113.67 (32.56)	0.261	121.58 (34.47)		106.65 (28.75)*	127.17 (34.58)*	**0.050**
**HDL, (mg/dl)**	48.15 (9.07)	46.43 (9.66)	47.56 (9.62)	0.766	48.90 (9.71)		46.08 (8.48)	46.40 (9.67)	0.404
**Cholesterol, (mg/dl)**	195.62 (41.23)	186.13 (26.78)	181.56 (37.96)	0.298	190.28 (37.62)		172.58 (30.43)*	197.33 (36.04)*	**0.022**
**TG, (mg/dl)**	101.74 (35.29)	113.83 (47.24)	101.67 (40.92)	0.422	106.30 (43.21)		99.23 (35.94)	111.48 (43.42)	0.569
**AIP**	−0.05 (0.21)	0.01 (0.25)	−0.05 (0.20)	0.518	−0.05 (0.24)		−0.04 (0.18)	0.00 (0.24)	0.672
**Glucose, (mg/dl)**	90.94 (12.21)	91.43 (11.78)	92.44 (8.88)	0.871	91.60 (11.62)		92.31 (9.36)	90.68 (12.13)	0.873
**Insulin, U/mL**	13.55 (7.98, 25.73)	16.20 (9.78, 25.70)	14.60 (9.80, 21.00)	0.882	15.25 (10.00, 23.98)		16.30 (9.78, 25, 80)	14.50 (8.75, 24.85)	0.728
**HOMA-IR**	3.41 (1.74, 5.66)	3.53 (2.15, 6.17)	3.42 (2.13, 5.03)	0.902	3.45 (2.24, 5.54)		3.65 (2.26, 5.80)	3.20 (1.80, 5.96)	0.868
**QUICKI**	0.33 (0.04)	0.32 (0.03)	0.32 (0.03)	0.776	0.32 (0.03)		0.32 (0.03)	0.33 (0.03)	0.764
**SBP (mmHg)**	116.15 (18.01)	114.43 (12.15)	112.44 (15.80)	0.582	115.03 (17.14)		112.15 (13.08)	116.04 (15.46)	0.565
**DBP (mmHg)**	75.35 (10.32)	79.67 (10.51)	75.67 (14.65)	0.270	77.20 (11.06)		73 81 (9.30)	79.52 (14.84)	0.196
**Mets (%)**	36.0	40.0	24.0	0.758	52.0		16.0	32.0	0.804
**CARTPT (%)**				0.800					0.865
CC	18.2	10.3	17.9		15.4		14.8	16.7	
CT	15.2	24.1	21.4		25.6		11.1	20.8	
TT	66.7	65.5	60.7		59.0		74.1	62.5	

Data are presented as mean (SD) or median (25 and 75 percentiles)

*Analysis of variance for continuous variables and χ^2^ test for categorical variables

*Abbreviations*: *BMI* Body mass index, *WC* Waist circumference, *SES* Socio-economic status, *HOMA-IR* homeostasis model assessment of insulin resistance, *LDL-C* Low density lipoprotein cholesterol, *HDL* High-density lipoprotein, *SBP* Systolic blood pressure, *DBP* Diastolic blood pressure, *TG* Triglyceride, *QUICKI* Quantitative insulin sensitivity check index, *AIP* Athrogenic indx of plasma

**Table 3:** Results from Structural Equation Modeling of relations between the CARTPT rs2239670 polymorphism, diet, socio-demographic and psychological variables and serum glycemic levels and lipid profile among individuals with obesity

Model Path	Standardized estimate^a^	SE	***P***
**Model 1** **Direct effects**			
Appetite → GL	1.356	0.463	0.003
Gender → TG	−24.740	8.516	0.004
Triglyceride → LDL-C	−0.200	0.001	0.000
HDL → LDL-C	−0.999	0.004	0.000
Cholesterol → LDL-C	1.001	0.001	0.000
Triglyceride → HDL	−0.0435	0.011	0.000
Gender → HDL	3.885	1.228	0.002
Age → Cholesterol	0.938	0.333	0.005
**Indirect effects via GI and GL**			
Age →LDL-C	0.917	0.332	0.006
Gender → HDL	1.047	0.484	0.031
**Model 2**			
**Direct effects**			
Appetite → GL	1.358	0.464	0.003
Stress → Insulin	0.007	0.002	0.001
Age → Insulin	0.006	0.003	0.023
**Model 3**			
**Direct effects**			
CARTPT→MetS	0.037	0.022	0.043
**Indirect effects via GI**			
CARTPT→ MetS	0.039	0.017	0.009

*Abbreviations*: *GI* Glycemic index, *GL* Glycemic load, *LDL* Low density lipoprotein, *HDL* High-density lipoprotein, *MetS* Metabolic syndrome, *CARTPT* Cocaine- and amphetamine-regulated transcript, *SE* Standard error of the estimate; All standardized path coefficients shown were significant (*P* < 0.05)

^a^Standardized path coefficients

**Table 4:** Goodness of fit indices for models

Model	DF	χ2	χ2 / DF	RMSEA	SRMR	CFI
1	24	47.973	1.99	0.075 (0.043–0.106)	0.072	0.985
2	12	22.879	1.90	0.072 (0.023–0.116)	0.039	0.901
3	6	7.678	1.28	0.055 (0.000–0.152)		0.997

χ^2^: Chi-Square value, *DF* Degrees of Freedom, *RMSEA* Root Mean Square Error of Approximation, *SRMR* Standardized Root Mean Square Residual, *CFI* Comparative Fit Index

1The final model with the best fit according to the values of several fit indices for the association between socio-demographic variables, diet and insulin resistance indices

2The final model with the best fit according to the values of several fit indices for the association between socio-demographic variables, diet and cardio-metabolic risk factors

**Table 5:** Total effects of genetic, socio-demographic and psychological parameters and diet on metabolic syndrome among adults with obesity using SEM

Model 3	Total		
	Standardized estimate^a^	SE^b^	*P*.value
GI → MetS	0.024	0.010	**0.008**
GL → MetS	0.004	0.005	0.181
CARTPT → MetS	0.037	0.022	**0.043**
Gender → GL	−15.376	10.809	0.077
Age → GL	0.093	0.639	0.442
PA → GL	6.183	5.390	0.125
SES → GL	−1.096	1.795	0.270
Stress → GL	0.670	0.459	0.072
Appetite → GL	0.255	0.415	0.269
CARTPT → GL	3.339	0.242	**0.000**
Gender → GI	−2.462	3.741	0.255
Age → GI	−0.195	0.175	0.133
PA → GI	1.988	1.645	0.113
SES → GI	−0.160	0.654	0.403
Stress → GI	0.045	0.143	0.377
Appetite → GI	0.099	0.149	0.253
CARTPT → GI	1.647	0.080	**0.000**

*Abbreviations*: *GI*, Glycemic index, *GL* Glycemic load, *SES* Socio-economic status, *MetS* Metabolic syndrome, *PA* Physical activity, *CARTPT* Cocaine- and amphetamine-regulated transcript, *SE* Standard error of the estimate

^a^Standardized path coefficients

^b^Total effect is defined as the sum of direct and indirect effects

## Discussion

To the best of our knowledge, this is the first study to simultaneously examine the direction and the relationships of socio-demographic, genetic, psychological and dietary parameters with cardio-metabolic risk factors and MetS in adults with obesity using the path analysis. Our main finding is that CARTPT rs2239670 polymorphism, not only by direct effect, but also can indirectly and through the mediation of dietary glycemic indices influence MetS. Additionally, GI score was positively related to MetS presence and also significant relationships were found between CARTPT variant and dietary glycemic indices (GI and GL). Similarly, higher dietary glycemic indices appeared to mediate the effects of some of socio-demographic factors (age and gender) on lipid profile. In addition to direct effects of the aforementioned factors on some of the lipid profile (cholesterol and HDL), we observed significant paths directly from the age and stress to the insulin.

In recent years, due to an increase in carbohydrate intake and changes in food processing, dietary GI and GL, as carbohydrate quality indicators, have enhanced [60]. Accumulating scientific evidence has shown that high-GI and -GL diets seem to enhance the risk of chronic diseases such as type2 diabetes, MetS, CVD, and certain types of cancers [61]. Although the significant positive association between GI and MetS presence observed in our study was in line with the aforementioned studies [62], the relationships between these indicators and MetS and its components are still controversial [18]. For instance, evidence from different randomized controlled trials (RCTs) approved the beneficial effects of low-GI or GL diets on triglyceride [63] or HDL concentrations [64]. In contrast, a recent meta-analysis has failed to find such associations [15]. The reason for these discrepant finding is not clear, but may be partly due to the difference in the population characteristics such as ethnicity and genetic backgrounds. Considering the role of genetic factors in the incidence of many diseases, the CARTPT rs2239670 variant was found to be associated with MetS presence among adults with obesity in present study. Despite the fact that the rs2239670 variant was directly related to the presence of MetS, indirect effect of this variant, through dietary GI on the MetS presence was also shown in our analysis. So, it appeared that the associations of this variant with variables of interest were mediated through higher dietary GI proposes the hypothesis that changes in the quality of carbohydrates consumed may be necessary as a recommendation for the prevention of MetS and cardio-metabolic risk factors.

Generally, most commonly used methods such as ANOVA or multiple regression techniques model individual observations, but SEM allows us to simultaneously assess all complex interrelationships amongst a number of potentially inter-dependent variables under a conceptual model by investigating all relevant regression pathways, including direct and indirect [24]. While no evidence is available on the direct and indirect associations of potential genetic and dietary factors with cardio-metabolic risk factors and MetS among adults with obesity in a multifactorial model and the current study is the only SEM modeling study, there were many studies have investigated the association of these indicators of dietary carbohydrate quality (GI and GL) [17] and also genetic factors with chronic diseases [65, 66]. In other words, the direct effects of glycemic indices on obesity and its-related health outcomes have been investigated using regression analysis (without examining indirect effects and using the SEM) and their results confirm the mediatory effects of these indicators which found in our analysis [17, 67, 68]. In fact, it seems that CARTPT may be linked to GI and GL [5] which in turn may leads to a greater probability of MetS presence. A recent study among Iranians reported that the quantity and quality of carbohydrate in the diet was positively associated with the risk of MetS and some of its components [69]. Although the mechanisms behind these effects are largely unknown, it has been suggested that high-GI diets may enhance hunger and lead to overeating and obesity [70]. As expected, we found a positive direct link between appetite and both dietary glycemic indicators. On the other hand, it has been proposed that the effects of high GI diets can be explained by reduced fiber intake such as resistant starch which may play a role in metabolism independent of their influences on postprandial glycaemia and insulin response [71].

The significant association which was found between CARTPT polymorphism and MetS presence in the current work was in agreement with earlier studies [8]. Likewise, several prior studies have identified polymorphisms in the CART gene of individuals with obesity [72] and it seems that any alterations in CARTPT are associated with reduced metabolic rate, hyperphagia, obesity and increased the risk of type II diabetes [73]. Nevertheless, the specific association between CARTPT rs2239670 polymorphism and obesity or its-related complications has rarely been examined and the only study in this regard, which was conducted in Malaysia, did not find any association between the CARTPT rs2239670 variant and obesity [74]. These contradictory findings suggest further research efforts in this regard among various populations.

The results pertaining to the positive indirect associations of age with LDL-C and gender with HDL are in accordance with previous studies [75]. It was shown that gender significantly modified the effects of glycemic index and glycemic load on cardio-metabolic risk factors, and these associations seemed to be the most evident in women than men [76]. For instance, Fan J et al. reported a positive association between the risk of cardiovascular disease and glycemic load in women, but not in men [76]. In spite of the most previous studies showing an inverse relationship between age and dietary glycemic indices [77], we observed an indirect effect of age on LDL-C which suggests that hormone-dependent effects and changes in diet and body composition may be reasons for age-related increment of LDL-C [78, 79]. It was also found in the present study that stress had a direct positive effect on insulin level, which is in agreement with many other studies [80]. Accordingly, a large body of animal studies has confirmed that stress has a role in the insulin secretion from isolated islets of Langerhans [81] and can result in insulin resistance in different tissues [82]. Such a finding is consistent with recent human studies [80]. It has been proposed that chronic psychological stress causes its effects via hyper-stimulation of the hypothalamic–pituitary–adrenal axis [83].

We detected a positive association between dietary GI and LDL-C concentration among male subjects while a similar association was found in relation to GL among female subjects. Similarly, another positive relationship was documented between dietary GL and cholesterol level in women. These results are in line with earlier studies that have reported differences in lipid profile between participants who consumed a high or a low GI diet [68]. For example, Levitan et al. reported that dietary GI was related to increases in LDL-C, LDL/HDL cholesterol ratio and TG [84].

As for strengths, according to our knowledge, this is the first time that the mediating effects of glycemic indices in the association between psychological and socio-demographic factors and genetic susceptibility to obesity and MetS have been examined with the use of the SEM approach. Additionally, we applied a reliable and validated FFQ for dietary assessment. Nonetheless, there are some limitations that need to be outlined. First, since this is a cross-sectional study, ascertained causality or temporality of associations cannot be argued, but, the results contribute to generate hypotheses that can then be tested by prospective studies. Second, due to relatively small sample size of our study, our observations should be interpreted with caution and these mediation models require to be replicated longitudinally. Third, our findings may not be extrapolated to all Iranian population, as this project was performed in Tabriz with different dietary intakes and lifestyle factors than other parts of the country. Fourth, underreporting of dietary intake especially in people with obesity is common which could cause misclassifications in dietary variables and null results [85]. To avoid this source of bias in our study, upper and lower extreme values of energy intake were excluded from the analysis. Fifth, although potential confounders were controlled, residual confounding might still exist. Last, since Iranian food glycemic index table includes only some limited local food items, international GI tables were used which could be a source of errors because the effect of variety, degree of ripeness, growing conditions, processing, and cooking may affect GI values.

In conclusion, findings from the structural equation models suggest a hypothesis of the mediating effect of glycemic indices in the relationship between genetic susceptibility to obesity and MetS presence. Moreover, a direct effect of CARTPT gene polymorphism was observed on outcome variable (MetS). In addition to the direct effects of demographic parameters on cardio-metabolic risk factors, indirect effects through the mediation of dietary glycemic indicators were found. Thus, it seems that focus on improving the quality and quantity of carbohydrate needs to be targeted in individuals with greater genetic predisposition to prevent MetS, and further investigations of this kind are required to be performed in large prospective studies to confirm the identified associations.

## Data Availability

Data of the current work is available through a reasonable request from corresponding author.
